# Dynamics of the COVID-19 epidemic in Ireland under mitigation

**DOI:** 10.1186/s12879-021-06433-9

**Published:** 2021-08-03

**Authors:** Bernard Cazelles, Benjamin Nguyen-Van-Yen, Clara Champagne, Catherine Comiskey

**Affiliations:** 1 UMMISCO, Sorbonne Université, Paris, France; 2 INRAE, Université Paris-Saclay, MaIAGE, Jouy-en-Josas, France; 3 Eco-Evolution Mathématique, IBENS, UMR 8197, CNRS, Ecole Normale Supérieure, Paris, France; 4 Swiss Tropical and Public Health Institute, Basel, Switzerland; 5 Universty of Basel, Basel, Switzerland; 6 School of Nursing and Midwifery, Trinity College Dublin, The University of Dublin, Dublin, Ireland

**Keywords:** COVID-19, Ireland, Stochastic model, Time varying parameters, Mitigation

## Abstract

**Background:**

In Ireland and across the European Union the COVID-19 epidemic waves, driven mainly by the emergence of new variants of the SARS-CoV-2 have continued their course, despite various interventions from governments. Public health interventions continue in their attempts to control the spread as they wait for the planned significant effect of vaccination.

**Methods:**

To tackle this challenge and the observed non-stationary aspect of the epidemic we used a modified SEIR stochastic model with time-varying parameters, following Brownian process. This enabled us to reconstruct the temporal evolution of the transmission rate of COVID-19 with the non-specific hypothesis that it follows a basic stochastic process constrained by the available data. This model is coupled with Bayesian inference (particle Markov Chain Monte Carlo method) for parameter estimation and utilized mainly well-documented Irish hospital data.

**Results:**

In Ireland, mitigation measures provided a 78–86% reduction in transmission during the first wave between March and May 2020. For the second wave in October 2020, our reduction estimation was around 20% while it was 70% for the third wave in January 2021. This third wave was partly due to the UK variant appearing in Ireland. In June 2020 we estimated that sero-prevalence was 2.0% (95% CI: 1.2–3.5%) in complete accordance with a sero-prevalence survey. By the end of April 2021, the sero-prevalence was greater than 17% due in part to the vaccination campaign. Finally we demonstrate that the available observed confirmed cases are not reliable for analysis owing to the fact that their reporting rate has as expected greatly evolved.

**Conclusion:**

We provide the first estimations of the dynamics of the COVID-19 epidemic in Ireland and its key parameters. We also quantify the effects of mitigation measures on the virus transmission during and after mitigation for the three waves. Our results demonstrate that Ireland has significantly reduced transmission by employing mitigation measures, physical distancing and lockdown. This has to date avoided the saturation of healthcare infrastructures, flattened the epidemic curve and likely reduced mortality. However, as we await for a full roll out of a vaccination programme and as new variants potentially more transmissible and/or more infectious could continue to emerge and mitigation measures change silent transmission, challenges remain.

**Supplementary Information:**

The online version contains supplementary material available at 10.1186/s12879-021-06433-9.

## Background

In the last months of 2019, grouped pneumonia cases were described in China. The etiological agent of this new disease, a betacoronavirus, was identified in January and named SARS-CoV-2. Meanwhile this novel coronavirus disease (COVID-19) spread rapidly from China across multiple countries worldwide. As of March 17, 2020, COVID-19 was officially declared a pandemic by the World Health Organization. COVID-19 has now spread throughout most countries causing causing millions of cases, killing hundreds of thousands of people and causing socio-economic damage [1]. Until vaccination campaigns are widely implemented, the expansion of COVID-19 with the appearance of newer, more transmissible and/or more infectious variants continue to threaten to overwhelm the healthcare systems of many countries.

The first case in Ireland was declared on the 29th of February 2020 followed by a rapid increase in reported infections leading to a peak in daily incidence in the week of April 10th to 17th. This peak was followed by a steady decline in daily cases reported until mid-August when a slow but steady increase in cases emerged. This increase was sustained and on Friday the 18th of September as a result of this increase the capital city, Dublin, was placed on a level 3 alert with movement restrictions and various lockdown measures. On September 25th a rural region in close proximity to the border of Northern Ireland was also placed on this level 3 alert [2].

Our aim is to examine the dynamics of the COVID-19 epidemic in Ireland using public data and a simple stochastic model. As occurs with the majority of epidemics, the COVID-19 epidemic has and continues to modify greatly during its course. Taking account of the time-varying nature of the different mechanisms responsible for disease propagation is always a major challenge. To tackle this aspect, we have used a previously proposed framework [3]. This framework uses diffusion models driven by fractional Brownian motion to model time-varying parameters embedded in a stochastic modified SEIR model, coupled with Bayesian inference methods. This mechanistic modeling framework enables us to reconstruct the temporal evolution of key parameters based only on the available data, under the non-specific assumption that it follows a basic stochastic process constrained by the observations. The advantages of this approach are the possibility of (i) considering all the specific mechanisms of the transmission of the pathogen (e.g. asymptomatic transmission), (ii) using different datasets simultaneously, (iii) accounting for all the uncertainty associated with the data used and, most importantly (iv) following the time-evolution of some of the key model parameters. This framework allows us to follow changes in disease transmission owing, for example, to Public Health interventions, which are of particular interest to us in the case the COVID-19 epidemic.

## Materials and methods

### Data

Large uncertainties are associated with the reported number of cases of COVID-19 [4, 5]. The lower number of reported cases is due to low detection and reporting rates, firstly because the testing capacity (RT-PCR laboratory capacity) was limited and has greatly varied during the course of this epidemic. Secondly, it is due to features of this new virus, such as transmission before the onset of symptoms and important asymptomatic transmission, which results in a low fraction of infected people attending the health facilities for testing.

This suggests that hospitalized data is likely to be the most accurate COVID-19 related data. Thus we mainly focus on hospitalized data published by the Health Protection Surveillance Centre (HPSC) [6]. We also mainly focus on incidence data to avoid all defects related to the use of cumulative data (see [7]), ie: daily hospitalized admission, daily ICU admission, daily deaths and daily hospital discharged. We also used “current bed used” both in hospital and in ICU as these are state variables of our model. Taking account of the large variability of the daily observations, since the 1st of June 2020 we have only used a weekly average of the daily values observed.

Since hospitalized data is only available from the 22th of March after the first mitigation measures (school closure) and that our aim was to model the dynamics of the epidemic before, during and after the NPI measures, we used daily incident infectious data available before the 25th of March. Nevertheless this data was associated with a low reporting rate and a large variance in the observational process used (see Inference part below).

### Model

**Fig. 1:**
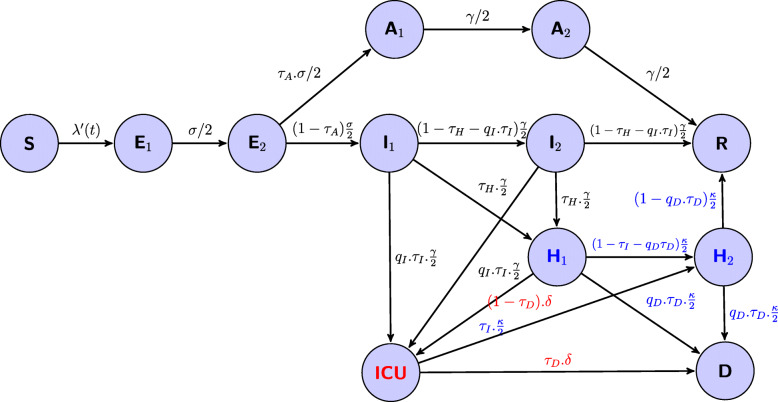
Flow diagram of the model, with *λ’(t) = β(t).(I*_*1*_ *+ q*_*1*_*.I*_*2*_ *+ q*_*2*_*.(A*_*1*_ *+ A*_*2*_*))/N* then the force of infection is *λ(t) = λ’(t).S(t)*. *β(t)* is the time-varying transmission rate, σ the incubation rate, *γ* the recovery rate, 1/*κ* the average hospitalized period, 1/δ the average time spent in ICU, *τ*_*A*_ the fraction of asymptomatics, *τ*_*H*_ the fraction of infectious hospitalized, *τ*_*I*_ the fraction of ICU admission, *τ*_*D*_ the death rate, *q*_*1*_ and *q*_*2*_ the reduction of transmissibility of *I*_*2*_ and *A*_*i*_, *q*_*I*_ the reduction of the fraction of people admitted in ICU and *q*_*D*_ the reduction of the death rate. The subscripts 1 and 2 are for the 2 stages of the Erlang distribution of the considered variable. The hospital discharge is the flow from H_2_ to R. Flows in blue are from hospital (H_i_) and flow in red from ICU

A simple model of extended stochastic Susceptible-Exposed-Infectious-Recovered (SEIR) also accounting for asymptomatic transmission and the hospital system has been developed (see eqs. A1-A3 in the *Supporting information* and Fig. 1). It is similar to others, which have been proposed to model and forecast the COVID-19 epidemic [8–11]. It includes the following variables: the susceptibles *S*, the infected non-infectious *E*, the infectious symptomatic *I*, the infectious asymptomatic *A*, the removed people *R*, and the hospital variables: hospitalized people *H*, people in intensive care unit *ICU*, hospital discharge *G*, and deaths at hospital *D*. We have also introduced Erlang-distributed stage durations (with a shape parameter equal to 2) for the *E*, *I*, *A* and *H* compartments to mimic a gamma distribution for stage duration in these compartments discounting inappropriate exponential stage durations (eqs. A1). As more and more people are being vaccinated in Ireland, the effect of vaccination is introduced in our model simply by considering the effect of vaccination on the depletion of susceptibles. For this, we removed from the susceptible compartment the “effectively protected vaccinated people” that are proportional to the number of people vaccinated with one and/or two doses (see eq. A2). The parameters are defined in Table 1 and in the *Supplementary information*.

**Table 1:** Defnition of the different parameters and their priors and posteriors based on current literature [8–11] (see also Fig. A1). For priors, some upper bound and/or lower bound have been imposed by the observations. U is for uniform distribution and tN for truncated normal distribution (tN [mean,std.,limit inf,limit sup])

Parameters	Definitions	Prior or constant value	Posterior Median, [95%CI]
*I*_*1*_*(0)*	Initial condition	U[5,100]	42, [18–81]
*S(0)*	Initial condition	*N* = 5,176,000	
*E*_*1*_*(0), E*_*2*_*(0), I*_*2*_*(0), A*_*1*_*(0), A*_*2*_*(0),*	Initial conditions	Use of steady-state conditions ^a^	
Other Initial Conditions	Initials conditions	0	
*β(0)*	Initial condition of the transmission rate	0.70	
*ν*	Volatility of the Brownian process	U[0.05,015]	0.133, [0.107–0.149]
*1/σ*	average duration of the incubation	tN[4,0.1,3,5]	3.99, [3.80–4.19]
*1/γ*	average duration of infectious period	tN[6,0.2,4.5,7.5]	6.00, [5.61–6.40]
*1/κ*	average hospitalized period	U [8, 20]	13.60, [12.15–15.15]
*1/δ*	average time in ICU	U [8, 20]	17.36, [14.86–19.57]
*τ*_*A*_	fraction of asymptomatics	U[0.30,0.70]	0.487, [0.310–0.685]
*τ*_*H*_	fraction of hospitalization	U[0.02,0.10]	0.027, [0.020–0.046]
*τ*_*I*_	fraction of ICU admission	U[0.05,0.15]	0.030, [0.023–0.045]
*τ*_*□*_	death rate	U[0.10,0.70]	0.411, [0.365–0.458]
*q*_*1*_	reduction of transmissibility	1.5. q_2_ but≤1	
*q*_*2*_	reduction of transmissibility	0.55 [8]	
*q*_*I*_	reduction of ICU admission fraction	0.10	
*q*_*D*_	reduction of the death rate	0.20	
*ρ*_*I*_	reporting rate for symptomatic infectious	U[0.02, 0.15]	0.092, [0.062–0.142]
*ρ*_*H*_	reporting rate for hospitalized people	U[0.95,1]	0.971, [0.951–0.997]
*ρ*_*ICU*_	reporting rate for the ICU admission	0.96	
*ρ*_*G*_	reporting rate for hospital discharge	0.96	
*ρ*_*D*_	reporting rate for death	0.98	

^a^ steady-state conditions are defined by: $\frac{d{E}_1}{dt}=\frac{d{E}_2}{dt}=\frac{d{I}_2}{dt}=\frac{d{A}_1}{dt}=\frac{d{A}_2}{dt}=0$

As the peaks of those hospitalized and those admitted to ICU are concomitant we consider that a weak fraction, *q*_*I*_*.τ*_*I*_ of infectious with severe symptoms goes directly to ICU. Even if the majority of deaths occur in the ICU, a small fraction, *q*_*D*_*.τ*_*D*_, can occur in hospital but not in intensive care.

An interesting sub-product of our framework is the possibility of estimating the time evolution of the effective reproduction number, *R*_*eff*_ [12]. *R*_*eff*_ is defined as the mean number of infections generated during the infectious period of a single infectious case at time *t*. It can be easily estimated using the steady-state form of a SEIR model. Taking into account the particularity of our model that considers different transmission capacity for different infectious, its value is a function of both the fraction of asymptomatic infectious *A*_*i*_*(t)*, τ_*A*_, and of symptomatic infectious *I*_*i*_*(t)*, 1-τ_*A*_:

$$ {R}_{eff}(t)=\left(\frac{\left(1+{q}_1\right)}{2}.\left(1-{\tau}_A\right)+{q}_2.{\tau}_A\right).\frac{\beta (t)}{\gamma }.\frac{S(t)}{N} $$

where *β(t)* is the transmission rate, *1/γ* is the infection duration, *τ*_*A*_ is the fraction of asymptomatic individuals in the population, (*1-τ*_*A*_) the proportion of symptomatic infectious individuals, *q*_*i*_ are the reduction in the transmissibility of some infected (*I*_*2*_) and asymptomatics (*A*_*i*_) and *N* is the population size.

### Inference

As we used Brownian process for modeling the time-varying transmission rate our model is stochastic, the likelihood is intractable and it is estimated with particle filtering methods (Sequential Monte Carlo, SMC). Then the particle filter is embedded in a Markov Chain Monte Carlo framework, leading to the particle Markov Chain Monte Carlo method (PMCMC) algorithm [13]. More precisely, the likelihood estimated by SMC is used in a Metropolis Hasting scheme (particle marginal Metropolis Hastings) (see *Supplementary information*). The priors of the inferred parameters are in Table 1.

For the inference the observations considered are daily incident infectious at the beginning of the epidemic, new hospitalized patients, new ICU admission, new deaths and hospitalized discharges. Hospital observations are only available after the lockdown (25th of March). Because these are count processes, we model their observations with Negative Binomial likelihoods (see *Supplementary information*). Current hospital data, observed, hospitalized patients (*H*_*1*_ *+ H*_*2*_ *+ ICU*) and ICU beds used (*ICU*) have also been used in the inference process and we make the assumption that these variables follow a normal distribution (see *Supplementary information*).

## Results

**Fig. 2:**
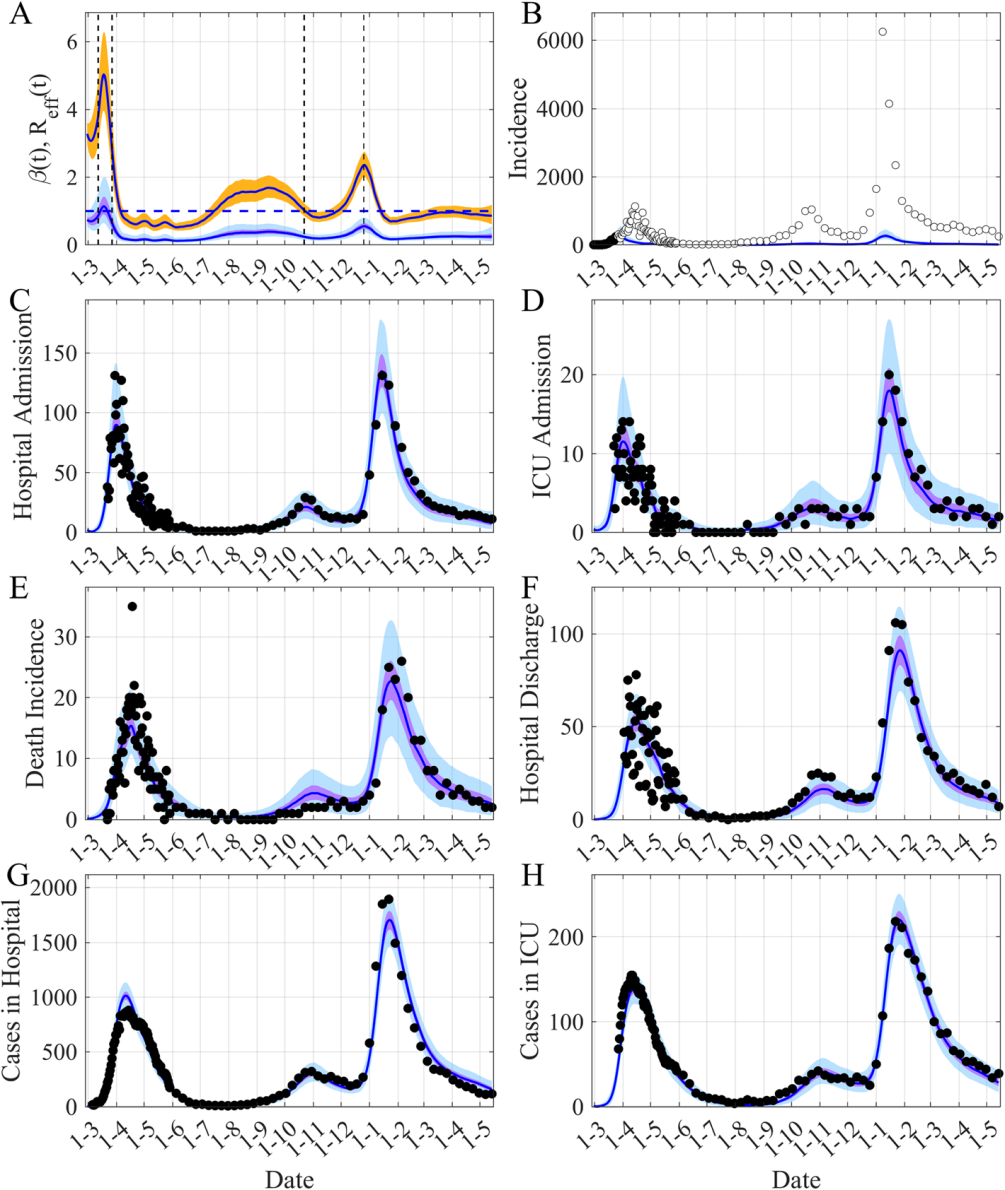
Reconstruction of the observed dynamics of COVID-19 in Ireland. **A** The time evolution of both *β(t)* and *R*_*eff*_ *(t)*. **B** Simulated observed daily incident infectious. **C**-**D** New daily admissions to hospital and to ICU. (**E**) Daily new deaths. **F** Hospital discharges. **G**-**H** Cases in Hospital and in ICU each day. The black points are observations used by the inference process, the white points are the observations not used. The blue lines are the median of the posterior of the simulated trajectories, the mauve areas are the 50% Credible Intervals (CI) and the light blue areas the 95% CI. In (**A**) the orange area is the 50% CI of *R*_*eff*_, the vertical dashed lines show the date of the main NPI measures and the horizontal dashed-line *R*_*eff*_ = 1. For all the graphs, the reporting rates are applied to the model trajectories (Fig. 3) as during the inference process for comparison to the observations

**Fig. 3:**
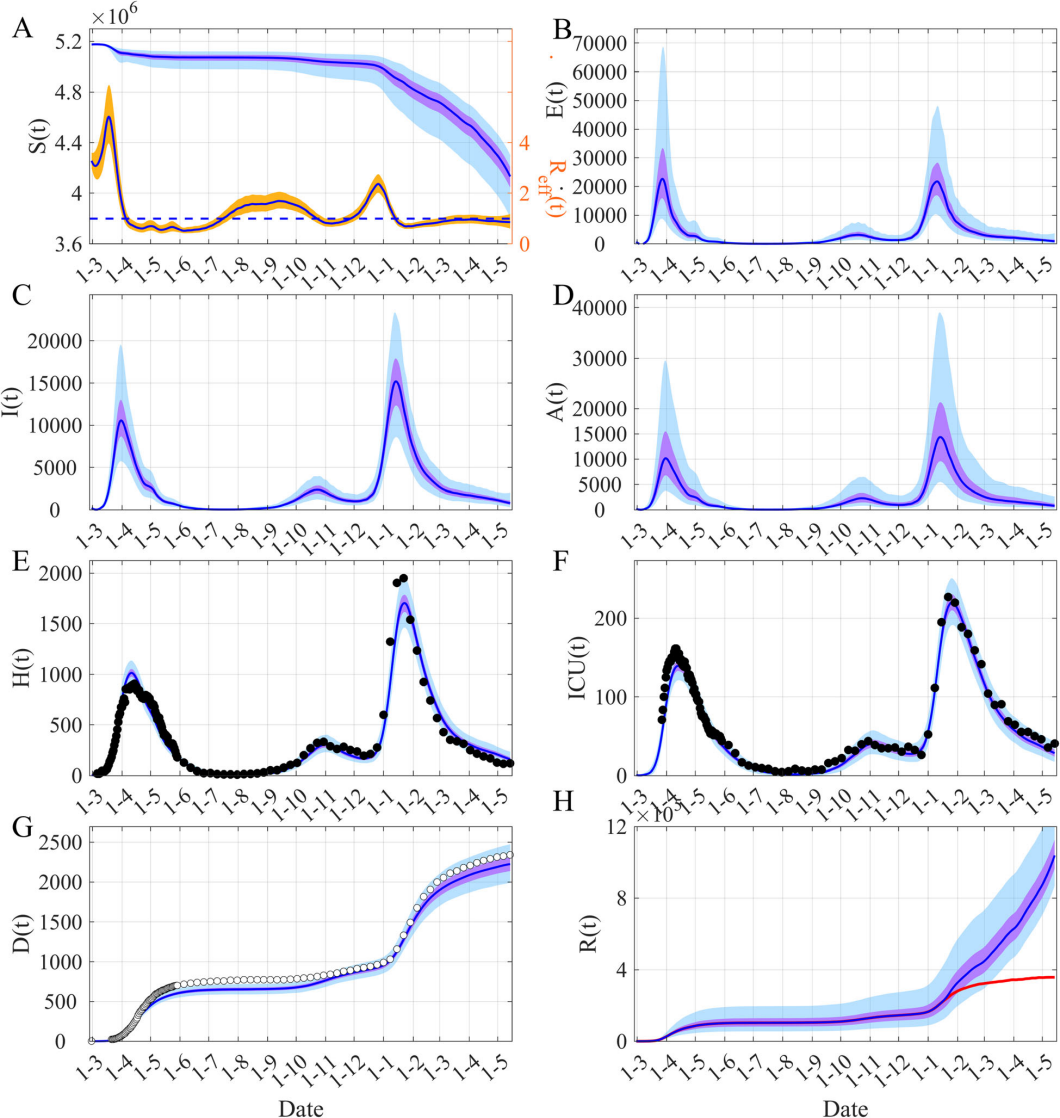
Dynamics of COVID-19 in Ireland. **A** Time evolution of both susceptibles *S(t)* and *R*_*eff*_ *(t)*. (**B**) Infected non infectious, *E(t) = E*_*1*_*(t) + E*_*2*_*(t)*. **C** Symptomatic infectious *I(t) = I*_*1*_*(t) + I*_*2*_*(t)*. **D** Asymptomatic infectious *A(t) = A*_*1*_*(t) + A*_*2*_*(t)*. (**E**) Hospitalized people *H(t) = H*_*1*_*(t) + H*_*2*_*(t) + ICU(t)*. **F** People in ICU, *ICU(t)*. **G** Cumulative death *D(t)*. (**H**) Removed *R(t)*. The blue lines are the median of the posterior of the simulated trajectories, the mauve areas are the 50% Credible Intervals (CI) and the light blue areas the 95% CI. In (**A**) the orange area is the 50% CI of *R*_*eff*_ and the horizontal dashed-line indicates *R*_*eff*_ = 1. In (**H**) the red line shows the median of *R(t)* when the “effectively protected vaccinated people” (see eq. A2) have been subtracted. The black points are observations used by the inference process, the white points are the observations not used

Figures 2 and 3 present our main results, Fig. 2 displays the fit of the model and Fig. 3 shows the dynamic of the model. The posteriors of the fitted parameters are in Table 1 and in Fig. A1.

Figure 2 illustrates the potential of the framework to effectively describe the numerous observations of this complex epidemic. The main characteristic this framework offers is the ability to reconstruct the time variation of the transmission rate *β(t)* (Fig. 2A) that is needed to fit the observations. We can then compute the time-variation of *R*_*eff*_ (Fig. 2A). The initial value of *R*_*eff*_ is around 3.2 in accordance with numerous published papers (e.g. [14]). The peak of *R*_*eff*_ around the time of the first hospital observations is presumably a compensation effect of the model to accommodate diverging trends between reported case data and hospital data. Then one can note a decrease of 78% of *R*_*eff*_ between the 1st of March and the 1st of May and a decrease of 86% between the 12th of March (school closure and lock down of offices, restrictions on travel etc) and the 1st of May (Fig. 2A). The reduction in the transmission following the second lockdown was around 20% (Fig. 2A). Nevertheless the reduction of *R*_*eff*_ was again significant (70%) for the large wave that was observed in January 2021, largely due to the UK variant [15, 16] (Fig. 2A). Given the temporality of the decline compared to the timing of the NPIs, these sharp decreases seem to be the result of the implementation of the mitigation measures.

Another important characteristic of this epidemic is the fact that the peak of daily hospital admission and daily ICU admission are concomitant (Figs. 2G-H), this concomitance has influenced the structure of the model we developed.

A final important point concerns the observed daily incident infectious. It is a source of data that the model has not taken into account in the inference process (Fig. 2B). We fit the model to the daily incident infectious up to March 25th only (black points on Fig. 2B), and plot our daily incident infectious estimates with the corresponding estimate of the reporting rate, with a median of 0.09 (95% CI: 0.06–0.14). These data highlight that the first peak in observed incident infectious comes 2–3 weeks late, and is higher than expected. This shows that it is important to take into account a delay in reporting, for instance using models for nowcasting [17, 18]. This also clearly illustrates that the reporting rate has greatly evolved during the course of the epidemic, with part of the increase maybe explained by a greater proportion of asymptomatics tested as time went on, whereas in the model the people tested are considered symptomatic. It is worth noting that as the epidemic progressed, after November 2020, the observed positive cases became more consistent with the hospital data (Fig. 2B-D).

Figure 3 displays the dynamic of the model. Figures 3C-D show that the asymptomatic infectious are as important as symptomatics but with a larger uncertainty due to lack of information available in the data. Indeed, the data used contain very little information on asymptomatics and we observe identical prior and posterior distributions for the rate of asymptomatic, *τ*_*A*_ (see Fig. A1).

**Fig. 4:**
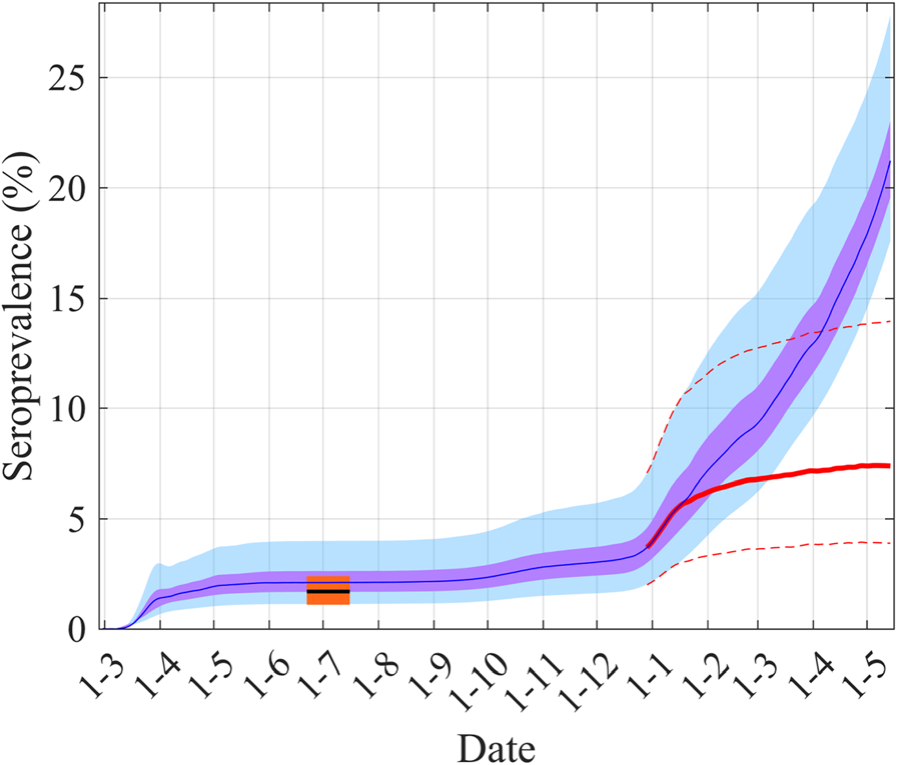
Estimation of the sero-prevalence and comparison with the value from a serological survey study [19]. The blue lines are the median of the posterior of the simulated trajectories, the mauve areas are the 50% CI and the light blue areas the 95% CI. The black line, around June–July, is for the median value of the serological survey, the orange area is for its 95%CI. The red line shows the median of sero-prevalence without the effect of vaccination simply by subtracting from the removed (*R(t)*) the “effectively protected vaccinated people” (see eq. A2) and the dashed red lines its 95%CI

Our model also allows us to estimate the sero-prevalence (Fig. 4). Our estimation for the 1st July is 2.1% (95% CI: 1.2–3.6%) and is in complete accordance with a survey study that shows a sero-prevalence of 1.7% (95% CI: 1.1–2.4%) between 22nd June and 16th July 2020 [19]. Figure 4 displays our estimation of the time evolution of the sero-prevalence that shows a large increase from the beginning of January 2020 due to the high propagation of the UK variant [15, 16] but, also, it would seem, due to the rolling out of the vaccination.

## Discussion

The need globally to accurately model COVID-19 mitigation strategies and asymptomatic transmission in order to plan for the burden on hospital admissions was identified early in the pandemic [20]. Davies et al. [21] within their models in the United Kingdom have predicted that extreme measures are probably required to prevent an excess of demand on hospital beds, especially those in ICUs during 2021. Similarly in France, Di Domenico et al. [9] have used modeling techniques calibrated with hospital admission data to model the impact of mitigation strategies to predict the scale of the epidemic within the Ile-de-France region. In the same way, we provide estimations of the dynamics of the COVID-19 epidemic in Ireland and its key parameters. The main characteristics of our approach is accounting for non-stationarity by embedding time-varying parameters in a stochastic model coupled with Bayesian inference. This mechanistic modeling framework enables us to reconstruct the temporal evolution of the transmission rate of the COVID-19 based only on the available data, under the non-specific assumption that it follows a basic stochastic process constrained by the observations. We can also describe the time evolving COVID-19 epidemic, quantifying the effects of mitigation measures on the virus transmission during and after the three waves suffered, and also estimate the sero-prevalence.

With our approach that mainly uses well-documented hospital data, we found a reduction of transmissibility of the SARS-CoV-2 of 78–86% after the implementation of the mitigation measures for the first wave. Our reduction estimations were around 20% for the second wave in October–November 2020 but more than 70% for the third wave in January–February 2021. These reductions in transmission may reflect the nature of the mitigation measures introduced in the country. For the second wave, these measures were less restrictive than during the first and third wave, nevertheless the second wave was also less severe. These results are in accordance with the results published on the effects of mitigation measures in Europe during the first wave [14, 22]. For example, Garchitorena et al. [22] by comparing 24 non-pharmaceutical interventions found that the median decrease in viral transmission was 74%, which is enough to suppress the epidemic and that a partial implementation of different measures resulted in lower than average response efficiency.

Our results also highlighted that the observed confirmed cases are only a small fraction of the total number of cases, only the tip of the iceberg (see [4]). This underlines that human behavior in the face of testing as well the delays in reporting, must be accounted for, for instance using models for now-casting [17, 18]. For example, in France it has been estimated that the detection rate increased from 7% in mid-May to 40% by the end of June, compared to well below 5% at the beginning of the epidemic [23]. Then data from hospital system published by health authorities are crucial for understanding the course of this epidemic. These data are well measured, but are observed with a delay in relation to contamination. Nevertheless, these delays can be easily account for by mathematical models.

Our study is not without limitations. Our model like all complex SEIR models developed for COVID-19 is non-identifiable which means that it is likely that several solutions exist and we only present one of the most likely. This point is always overlooked but see Li et al. [8]. The major limitation is the use of the classical homogeneous mixing assumption in which all individuals are assumed to interact uniformly and ignores heterogeneity between groups by sex, age, geographical region. In all cases taking an age structure and mixing matrix appears insufficient and heterogeneity of contact is important (see [24]). However this kind of data is not easily available. Another weakness is perhaps the neglect of age-structure in the model to simulate age-based predictions as we enter the time of children returning to school. These weaknesses are however a future research development given the performance of the current model. Nevertheless in our opinion, these limitations are compensated for taking non-stationarity of this epidemic into account and by the fact that our results are mainly driven by hospital data, which is more accurate than the number of infected cases. Precise data from serological studies at different time periods would significantly reduce the uncertainties of the model predictions [25, 26].

The key strength of the current Irish study is the fit of the model to the current observed data on hospitalizations, deaths and ICU cases that were likely to be the most accurate COVID-19 related data [27]. This allows us to present the first Irish modeling estimates of sero- prevalence. The model presented predicted that in Ireland as of the 1st July 2020 between 1.2 and 3.5% of the population had been infected either as a symptomatic or asymptomatic case. This is in complete accordance with preliminary national serological results, which found that among 12 to 69 year olds living in Ireland the sero-prevalence rate was estimated between 26th June and 20th July 2020 at 1.7% (95% CI: 1.1–2.4%) [19]. Due to the high number of infected people during the second wave and especially during the third wave, by mid-May 2021, the sero-prevalence was estimated to be greater than 20%. This high value also reflects the result of the rolling out of the national vaccination programme (Fig. 4).

For the first wave, our sero-prevalence predictions contrast with those of more densely populated areas. For the first wave, estimated serological prevalence in the United Kingdom based on a random sample of home based testing has found that 6.0% (95% CI: 5.8–6.1%) of individuals tested positive, of these one third (32.2%, (95% CI, 31.0–33.4%)) reported no symptoms and were asymptomatic [21, 28]. Overall the authors estimated that 3.36 million (3.21 million to 3.51 million) people had been infected with SARS-CoV-2 in England by the end of June 2020. This estimate was substantially higher than the recorded numbers in the UK of 315,000 cases. This is in accordance with observations from Spain where between April and May 2020, sero-prevalence was 5% and only few cases of these people had a PCR test [29].

Undocumented infections particularly asymptomatic infections are known to be the silent drivers of infection. Many studies [29–35] that have investigated the impact of asymptomatic carriers on COVID-19 transmission state that, in a public health context, the silent threat posed by the presence of asymptomatic carriers in the population results in the COVID-19 pandemic being much more difficult to control. These studies show that the population of individuals with asymptomatic COVID-19 infections is contributing to driving the growth of the pandemic. Li et al. [8] estimate that in the early stages of the epidemic in China 86% of all infections were undocumented (95% CI: 82–90%). However perhaps what is more important according to Li et al. [8] was that the transmission rate of undocumented infections per person was 55% the transmission rate of documented infections (95% CI: 46–62%), yet, because of their greater numbers, undocumented infections were the source of 79% of the documented cases. In Ireland, we can see from Fig. 3 that our model estimates that the number of asymptomatic infectious is of the same order of magnitude as the number of symptomatic infectious but with a larger uncertainty. This highlights that there is not enough information in the data to go beyond the published values that have been considered in the prior of *τ*_*A*_. It also emphasizes the importance of asymptomatic transmission, which is very difficult to observe. However, considering this large uncertainty, the computation of the part of asymptomatic transmission is not relevant.

We also found other interesting results such as a significant similarity between the trend of mobility and our estimation of the transmission between the epidemic waves (see Fig. A2 and [36]), highlighting the importance of following the evolution of mobility when relaxing mitigation measures to anticipate the future evolution of the spread of the SARS-CoV-2 [37].

## Conclusions

In this work we have used a stochastic framework that accounts for the time-varying nature of the COVID-19 epidemic by using time-varying parameters and hospital data to provide a description of this evolving epidemic. Our results demonstrate that Ireland has significantly reduced transmission by employing mitigation measures, physical distancing and and long lockdowns for wave 3. This has avoided the saturation of healthcare infrastructures, flattened the epidemic curve during each wave and likely greatly reduced mortality. Our framework that accounts for the non-stationarity of the transmission also offers the possibility of computing the time varying *R*_*eff*_*(t)* and then to offer an interesting tool to follow the evolution of the COVID-19 epidemic. This tool could prove particularly useful in analyzing this new phase of this special epidemic, as new variants potentially more transmissible and/or more infectious could continue to emerge and mitigation measures change silent transmission.

## Supplementary Information

**Additional file 1: **Model equation, Bayesian inference, Prior and Posterior distributions, **Figs. A1-A2**. **Supplementary Fig. A1.** Prior and posterior distributions for the model inferences presented Fig. 2. *I*_*1*_(0) initial value of infectious, *ν* is the volatility of the Brownian process of *β*(*t*), 1/*σ* the average duration of the incubation, 1/*γ* the average duration of infectious period, 1/*κ* the average hospitalized period, 1/*δ* the average time spent in ICU, *τ*_*A*_ the fraction of asymptomatics, *τ*_*H*_ the fraction of infectious hospitalized, *τ*_*I*_ the fraction of ICU admission, *τ*_*D*_ the death rate, *ρ*_*I*_ the reporting rate for the infectious, *ρ*_*H*_ the reporting rate for the hospitalized people. The blue distributions are the priors and the discrete histograms are the posteriors. **Supplementary Fig. A2.** Parallel trends in our estimated *R*_*eff*_*(t)* (black lines) and Google Mobility (https://www.google.com/covid19/mobility/), retail and recreation mobility (continuous blue line) and transport mobility (dashed blue line) in Ireland. The mobility time series have been smoothed using moving average over a 7 days window. The vertical black dashed lines correspond to the start dates of the main mitigation measures.

## Data Availability

All surveillance data are available at the site from Health Protection Surveillance Centre (HPSC): https://www.hpsc.ie/a-z/respiratory/coronavirus/novelcoronavirus/casesinireland/epidemiologyofcovid-19inireland/ or https://covid19ireland-geohive.hub.arcgis.com/ The data used and the code are at https://www.dropbox.com/s/n0hi5syu80nup5a/ssm_SEIAR_Ireland.zip?dl=0.

## Abbreviations

HPSC: Health protection surveillance centre
ICU: Intensive care units
NPI: Non-pharmaceutical interventions
PMCMC: Particle markov chain monte carlo
RT-PCR: Reverse transcription polymerase chain reaction
SEIR: Susceptible exposed infectious recovered
SMC: Sequential monte carlo
