# Supplementary material for: Dynamics of the COVID-19 epidemic in Ireland under mitigation
Source: BMC Infect Dis. 2021 Aug 3;21:735. doi: 10.1186/s12879-021-06433-9 (PMC8329614; doi:10.1186/s12879-021-06433-9)
Supplement: Supplementary file 1 — Additional file 1: Model equation, Bayesian inference, Prior and Posterior distributions, Figs. A1-A2. Supplementary Fig. A1. Prior and posterior distributions for the model inferences presented Fig. 2. I1(0) initial value of infectious, ν is the volatility of the Brownian process of β(t), 1/σ the average duration of the incubation, 1/γ the average duration of infectious period, 1/κ the average hospitalized period, 1/δ the average time spent in ICU, τA the fraction of asymptomatics, τH the fraction of infectious hospitalized, τI the fraction of ICU admission, τD the death rate, ρI the reporting rate for the infectious, ρH the reporting rate for the hospitalized people. The blue distributions are the priors and the discrete histograms are the posteriors. Supplementary Fig. A2. Parallel trends in our estimated Reff(t) (black lines) and Google Mobility (https://www.google.com/covid19/mobility/), retail and recreation mobility (continuous blue line) and transport mobility (dashed blue line) in Ireland. The mobility time series have been smoothed using moving average over a 7 days window. The vertical black dashed lines correspond to the start dates of the main mitigation measures. [file 12879_2021_6433_MOESM1_ESM.docx]

**Dynamics of the COVID-19 epidemic in Ireland under mitigation**

Bernard Cazelles^1,2,3^, Benjamin Nguyen-Van-Yen^3^, Clara Champagne^4,5^ and Catherine Comiskey^6^

1. UMMISCO, Sorbonne Université, Paris, France

2. INRAE, Université Paris-Saclay, MaIAGE, Jouy-en-Josas, France

3. Eco-Evolution Mathématique, IBENS, UMR 8197, CNRS, Ecole Normale Supérieure, Paris, France

4. Swiss Tropical and Public Health Institute, Basel, Switzerland

5. Universty of Basel, Basel, Switzerland

6. School of Nursing and Midwifery, Trinity College Dublin, The University of Dublin, Dublin, Ireland

**Supplementary information**

***Model equation***

Our model is an extended stochastic SEIR model also accounting for asymptomatic transmission and the hospital system. It includes the following variables the susceptibles *S*, the infected non-infectious *E*, the infectious *I*, the removed people *R*, and the hospital variables: hospitalized people *H*, people in intensive care unit *ICU*, cured people *G*, and deaths at hospital *D*. We have also introduced Erlang-distributed stage durations (with a shape parameter equals 2) for the *E*, *I, A* and *H* compartments, for not taking into account inappropriate exponential stage durations. As more and more people have been vaccinated and as the duration of the immunity conferred shows a good persistence of the antibodies induced (Widge et al, 2021), the effect of vaccination is introduced simply by considering the effect of vaccination on the depletion of susceptibles. The differential equations below describe the deterministic version of our model, however it is important to note that we have used its stochastic version:


 (A1)

In the model *σ* is the incubation rate, *γ* the recovery rate, 1/*κ* the average hospitalized period, 1/*δ* the average time spent in ICU, *τ_A_* the fraction of asymptomatics, *τ_H_* the fraction of infectious hospitalized, *τ_I_* the fraction of ICU admission, *τ_D_* the death rate, *q_1_* and *q_2_* reduction of transmissibility. As the peaks of those hospitalized and those admitted to ICU are concomitant we consider that a weak fraction, *q_I_,.τ_I_* of infectious with severe symptoms go directly to ICU. Even if the majority of deaths occur in the ICU, a small fraction, *q_D_.τ_D_*, can occur in hospital but not in intensive care. Then *q_I_* and *q_D_* are the reduction of admission in ICU and of death rate, respectively.

*V* is linked to vaccinated people and can be considered as “effectively protected vaccinated people”. *V* is proportional to the number of people vaccinated with one and/or two doses as:

 (A2)

where *V_dosei_* is the population vaccinated with one or two doses, *d_e_* the delay after vaccination and *e_i_* is the vaccine efficiency. For the vaccine efficiency, the range documented in the literature is between [17%-73%] for one dose and [52 %-93%] for 2 doses (Bernal et al, 2021; Dagan et al, 2021; Hall et al, 2021; Moustsen-Helms et al, 2021; Thompson et al, 2021). Of course, the vaccine efficiency depends on population analyzed, the vaccine used and the delay after vaccination. This delay varies according to the studies as being between 1 week and 5 weeks (Bernal et al, 2021; Dagan et al, 2021; Hall et al, 2021; Moustsen-Helms et al, 2021; Thompson et al, 2021). Taking these analyses into consideration we used conservative values: *e_1_ =* 0.45; *e_2_ =* 0.85 and *d_e_ =* 14 days.

The main characteristic of our approach is the time-varying transmission rate *β(t)* that follows a Brownian diffusion process:

 (A3)

where ** is the volatility of the Brownian process (*dB*) that will be estimated during the fitting process. The logarithm transformation avoids negative values, which have no biological meaning.

***Bayesian inference***

Our inference is mainly based on incidence observations. Incidences used are described by the following equations:

 (A4)

Due to the use of a diffusion equation (A2) for the dynamic of the time-varying parameters, the model is stochastic. Thus equations (A1-A3) are considered in a stochastic framework solved with the Euler-Maruyama algorithm (Kloeden and Platen, 1999) implemented in the SSM platform (Dureau et al, 2013b).

Our model trajectories are related to the observed incidence using a negative binomial observation model, which is commonly used as observation model (Bretó et al, 2009). The observed incidences, *C_k,obs_(t)*, are assumed to be drawn from a negative binomial distribution with mean *ρ_k_C_k_(t)* and variance

*ρ_k_C_k_(t) + ϕ_k__k_ .C_k_(t))^2^* where *C_k_(t)* the incidence of the variable *i* simulated by the stochastic version of equations (A3), *ρ_i_* the reporting rates quantifying the amount of non reported incidences (see Table 1) and *ϕ_i_* are the overdispersion parameters.

Current hospital data, *H_obs_(t)* (corresponding to *H_1_+H_2_+ICU*) and *ICU_obs_(t)* (corresponding to *ICU*), are also available and therefore used to fit the model. We make the assumption that these variables *X_k,obs_* follow a normal distribution with fixed standard deviation proportional to its estimated higher value and with mean equals to the predicted values by the model *X_k_* (A2) times an observation rate *ρ_k_* (see Table 1)

For inference we have used Bayesian technics that need the computation of the likelihood. Since our epidemiological model is considered in a stochastic framework, their likelihood is intractable and it is estimated with particle filtering methods (Sequential Monte Carlo, SMC). With a given set of parameters, the SMC algorithm reconstructs sequentially the trajectory of the state variables and the time-varying parameters, and computes the associated likelihood. Firstly, the distribution of the initial conditions of the system is approximated with a sample of particles. Then, at each iteration, the particles are projected according to the propagation model up to the next observation point, they receive a weight reflecting the quality of their prediction compared to the observation, and the total likelihood is updated. A resampling step using the weights is performed before the next iteration, in order to discard the trajectories associated with low weight particles.

In order to estimate the parameters of the system, the particle filter is embedded in a Markov Chain Monte Carlo framework, leading to the PMCMC algorithm (Andrieu et al, 2010). More precisely, the likelihood estimated by SMC is used in a Metropolis Hasting scheme (particle marginal Metropolis Hastings) (Andrieu et al, 2010). The proposal distribution is a Gaussian whose co-variance matrix is adapted following the framework described in Dureau et al (2013).

With this PMCMC algorithm we have used uniform and truncated gaussian priors based on the current literature. The priors of the inferred parameters are in Table 1 and Fig. A1.

***Prior and Posterior distributions***

The Figure A1 displays prior and posterior distributions involved in our inference process.

***References***

Andrieu, C., Doucet, A., Holenstein, R. (2010). Particle Markov chain Monte Carlo methods. *Journal of the Royal Statistical Society: Series B*, 72, 269–342.

Bernal, J. L., Andrews, N., Gower, C., Stowe, J., Robertson, C., Tessier, E., ... & Ramsay, M. (2021). Early effectiveness of COVID-19 vaccination with BNT162b2 mRNA vaccine and ChAdOx1 adenovirus vector vaccine on symptomatic disease, hospitalisations and mortality in older adults in England. *MedRxiv*, 2021.03.01.21252652

Bretó, C., He, D., Ionides, E.L., King, A.A. (2009). Time Series Analysis via Mechanistic Models. *The Annals of Applied Statistics*, 3, 319-348.

Dagan, N., Barda, N., Kepten, E., Miron, O., Perchik, S., Katz, M. A., ... & Balicer, R. D. (2021). BNT162b2 mRNA Covid-19 vaccine in a nationwide mass vaccination setting. *New England Journal of Medicine*, 384, 1412-1423.

Dureau, J., Ballesteros, S., Bogich, T. (2013). SSM: Inference for time series analysis with State Space Models. https://github.com/JDureau/ssm/blob/master/doc/doc.pdf.

Hall, V. J., Foulkes, S., Saei, A., Andrews, N., Oguti, B., Charlett, A., ... & Atti, A. (2021). Effectiveness of BNT162b2 mRNA Vaccine Against Infection and COVID-19 Vaccine Coverage in Healthcare Workers in England. Multicentre Prospective Cohort Study (the SIREN Study), *The Lancet Preprints*. Available at SSRN: https://ssrn.com/abstract=3790399 or http://dx.doi.org/10.2139/ssrn.3790399

Kloeden, P. & Platen, E. (1999). Numerical Solution to Stochastic Differential Equations. Springer.

Moustsen-Helms, I. R., Emborg, H. D., Nielsen, J., Nielsen, K. F., Krause, T. G., Molbak, K., ... & Valentiner-Branth, P. (2021). Vaccine effectiveness after 1st and 2nd dose of the BNT162b2 mRNA Covid-19 Vaccine in long-term care facility residents and healthcare workers–a Danish cohort study. *MedRxiv*, 2021.03.08.21252200.

Thompson, M. G., Burgess, J. L., Naleway, A. L., Tyner, H. L., Yoon, S. K., Meece, J., ... & Gaglani, M. (2021). Interim estimates of vaccine effectiveness of BNT162b2 and mRNA-1273 COVID-19 vaccines in preventing SARS-CoV-2 infection among health care personnel, first responders, and other essential and frontline workers—eight US locations, December 2020–March 2021. *Morbidity and Mortality Weekly Report*, 70, 495-500.

Widge, A. T., Rouphael, N. G., Jackson, L. A., Anderson, E. J., Roberts, P. C., Makhene, M., ... & Beigel, J. H. (2021). Durability of responses after SARS-CoV-2 mRNA-1273 vaccination. *New England Journal of Medicine*, 384(1), 80-82.

**Figure A1**. Prior and posterior distributions for the model inferences presented Fig. 2. *I_1_*(0) initial value of infectious, ** is the volatility of the Brownian process of *β*(*t*), 1/*σ* the average duration of the incubation, 1/*γ* the average duration of infectious period, 1/*κ* the average hospitalized period, 1/*δ* the average time spent in ICU, *τ_A_* the fraction of asymptomatics, *τ_H_* the fraction of infectious hospitalized, *τ_I_* the fraction of ICU admission, *τ_D_* the death rate,* ρ_I_* the reporting rate for the infectious, *ρ_H_* the reporting rate for the hospitalized people. The blue distributions are the priors and the discrete histograms are the posteriors.

**Figure A2.** Parallel trends in our estimated *R_eff_(t)* (black lines) and Google Mobility (<https://www.google.com/covid19/mobility/>), retail and recreation mobility (continuous blue line) and transport mobility (dashed blue line) in Ireland. The mobility time series have been smoothed using moving average over a 7 days window. The vertical black dashed lines correspond to the start dates of the main mitigation measures.
